# Designing rapid-cycling, compact architecture in tomato for vertical farming

**DOI:** 10.1186/s43897-026-00248-5

**Published:** 2026-08-07

**Authors:** Yoonseo Lim, Jeong-Tak An, So Jin Park, Yeeun Han, Ho-Young Jeong, Chanhui Lee, Soon Ju Park, Dae-Hyun Jung, Choon-Tak Kwon

**Affiliations:** 1 Graduate School of Green-Bio Science, Kyung Hee University, Yongin, 17104 Republic of Korea; 2 Department of Smart Farm Science, Kyung Hee University, Yongin, 17104 Republic of Korea; 3 Interdisciplinary Program in IT-Bio Convergence System, Kyung Hee University, Yongin, 17104 Republic of Korea; 4 Department of Plant & Environmental New Resources, Kyung Hee University, Yongin, 17104 Republic of Korea; 5 Department of Seed R&D, ToolGen Inc, Cheongju, 28160 Republic of Korea; 6 Division of Applied Life Science, Plant Molecular Biology and Biotechnology Research Center, Gyeongsang National University, Jinju, 52828 Republic of Korea

Vertical farming enables stable production by maximizing yield per volume and decoupling crops from weather, water, and land constraints (Erekath et al. 2024). Leafy greens fit these systems because they mature quickly and occupy little space (Van Delden et al. 2021). By contrast, fruit crops are large, slow to mature, and poorly suited to stacked cultivation (Kwon 2023). A solution is to reconfigure shoot architecture to keep plants compact while sustaining reproductive output (SharathKumar et al. 2020). For example, tomato *self-pruning* (*sp*), *sp5g*, and *slerecta* (*sler*) “triple-determinate” plants show determinate growth, rapid fruiting, and shortened internodes, illustrating how architectural adjustment can improve agronomic traits in confined environments (Kwon et al. 2020). Although triple-determinate plants are compact, further improvement is needed for vertical farming (Lim et al. 2025). Gibberellin (GA) metabolism offers a tractable entry point for architectural tuning (Shani et al. 2024). Mutations in *GA 20-oxidase* (*GA20ox*) and *GA 3-oxidase* (*GA3ox*) often shorten internodes and produce compact architecture (Yu et al. 2025; Phillips et al. 2025). Yet excessive size reduction can diminish per-plant yield, underscoring the need for cultivars optimized for both compact growth and robust yield (Lim et al. 2025). Achieving this balance within multi-layer controlled-environment vertical farms remains a key challenge.

To address this challenge, we used a genome-editing strategy with trait stacking to convert an indeterminate tomato cultivar into a compact, high-density ideotype optimized for vertical farming (Fig. 1A). This architectural redesign combined rapid shoot growth termination, earlier flowering for faster crop cycling, shortened internodes, and maintenance of high yield potential (Fig. 1A). To further enhance the previously developed triple-determinate background, we identified additional candidate genes. Among the five *SlGA3ox* genes in tomato, *SlGA3ox2* showed the highest expression, we thus selected it as the editing target (Fig. 1B) (Lim et al. 2025). Using CRISPR–Cas9 with three guide RNAs in the triple-determinate background, we generated two independent homozygous *slga3ox2* alleles carrying frameshift mutations that introduced premature stop codons (Fig. S1A). After confirming phenotypic consistency between the two alleles (Fig. S1B-E), we evaluated the performance of the resulting quadruple mutant (*sp sp5g sler slga3ox2*^*CR−1*^; hereafter referred to as *slga3ox2*) in two hydroponic systems, namely a single-layer ebb-and-flow system in the greenhouse and a four-layer nutrient film technique (NFT) system in a vertical farm (Fig. 1C; Fig. S2A).

**Fig. 1:**
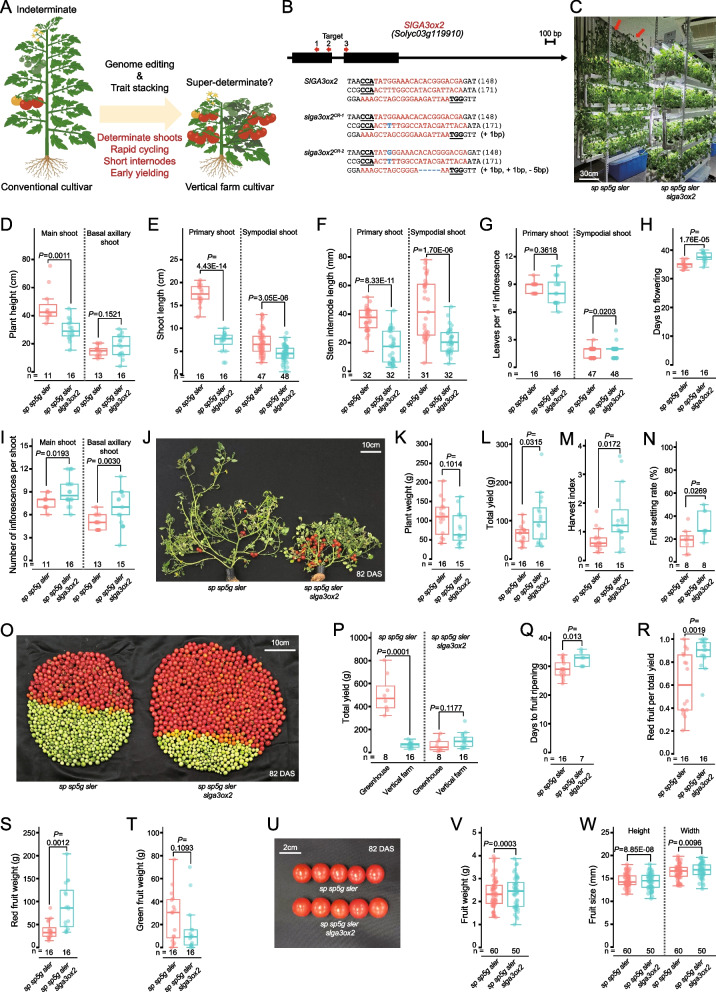
Development of compact and early yielding cherry tomato cultivar for vertical farming. **A** Schematic strategy to generate a vertical-farm type cultivar using conventional indeterminate cultivar. **B** Target sites of *SlGA3ox2* edited by CRISPR-Cas9 and representative mutant alleles. Red letters indicate guide RNA sequences, and blue letters represent DNA insertions and deletions. **C** Triple-determinate and *slga3ox2* plants grown under vertical farm conditions. The red arrows indicate shoots protruding beyond the cultivation space. **D-I** Quantification of plant height, shoot length, stem internode length, number of leaves up to the first inflorescence, days to flowering, and inflorescence number of main and basal axillary shoots. **J** Shoots of triple-determinate and *slga3ox2* plants at 82 days after sowing (DAS). **K-N** Plant weight, total yield, harvest index, and fruit setting rate at the first inflorescence of triple-determinate and *slga3ox2* plants. **O** Total fruits of triple-determinate and *slga3ox2* plants harvested from one layer of the vertical farm. **P** Comparison of total yield between triple-determinate and *slga3ox2* plants grown in greenhouse and vertical farm conditions. **Q** Days from flowering to fruit ripening of triple-determinate and *slga3ox2* plants. **R-T** Proportion of red fruits per total yield, red fruit weight, and green fruit weight of triple-determinate and *slga3ox2* plants. **U-W** Representative fruit phenotype, fruit weight, and fruit size of triple-determinate and *slga3ox2* plants. **D-I, K-N, P–T, V, W** Box plots show median, interquartile range, and whiskers; individual dots represent biological replicates. Numbers below each panel indicate sample size. Statistical significance was assessed by Student’s *t*-test, with *p* values shown in each panel

First, we performed phenotypic evaluation using an ebb-and-flow hydroponic system prior to cultivation and trait assessment in the vertical farm (Fig. S2). Compared to triple-determinate plants, *slga3ox2* plants exhibited significant reductions in plant height, shoot length, and internode length (Fig. S2B-G). The *slga3ox2* plants also showed earlier emergence of the first inflorescence, while the number of leaves per sympodial shoot remained unchanged (Fig. S2H, I). Although *slga3ox2* mutations altered stem elongation and flowering time, the number of inflorescences per shoot was comparable between the two genotypes (Fig. S2J-L). These results indicate that *slga3ox2* mutations promote compact growth and early flowering without negatively affecting inflorescence initiation. Next, we conducted a yield trial using ebb-and-flow hydroponics to compare triple-determinate and *slga3ox2* plants (Fig. S3). The reduction in plant size persisted until harvest, with *slga3ox2* plants exhibiting approximately 89.2% lower biomass than triple-determinate plants (Fig. S3A-C). This reduction in vegetative biomass proportionally affected total yield, resulting in an 87.5% decrease in total fruit yield per plant (Fig. S3B, D). Notably, at the harvest stage, *slga3ox2* plants exhibited a higher proportion of red fruits relative to green unripe fruits compared to the triple-determinate plants (Fig. S3E–G), reflecting differences in fruit color distribution at harvest. The harvest index showed no significant difference between two genotypes, indicating that reduced plant size rather than reproductive efficiency accounted for the yield reduction (Fig. S3H). Finally, individual fruit weight and size were both lower in *slga3ox2* plants compared to triple-determinate plants (Fig. S3I-K).

Under greenhouse ebb-and-flow hydroponics, *slga3ox2* plants produced less fruit per plant but displayed a markedly compact habit, supporting their suitability for space-limited vertical systems. Key architectural traits, such as plant height, primary and sympodial shoot length, and internode length, were again reduced relative to triple-determinate plants in the vertical farm using the NFT system, although the magnitude of reduction was smaller than in the ebb-and-flow trial (Fig. 1D-F; Fig. S2B-G). Although the number of leaves to the first inflorescence did not differ between genotypes in the vertical farm, *slga3ox2* plants flowered slightly later than the triple-determinate plants (Fig. 1G, H). In addition, *slga3ox2* plants exhibited an increased number of inflorescences per shoot under vertical farm conditions, a trend not observed under ebb-and-flow hydroponic cultivation (Fig. 1I; Fig. S2J-L). Collectively, these comparisons indicate that cultivation environment modulates *slga3ox2* phenotypes and underscore that optimizing the cultivation system is as critical as optimizing plant traits through genetic variation.

Consistent with the architectural findings, yield performance in the vertical farm differed from that in ebb-and-flow hydroponics. In the vertical farm, *slga3ox2* plants had biomass comparable to triple-determinate plants, in line with the milder size reduction observed under this system (Fig. 1D, J, K). Notably, *slga3ox2* plants showed slightly higher total fruit yield, harvest index, and fruit setting rate relative to triple-determinate plants (Fig. 1L-O). While *slga3ox2* yield did not differ significantly between the two cultivation systems, triple-determinate yield declined by roughly 80% in the vertical farm compared with ebb-and-flow system in the greenhouse (Fig. 1P). Thus, the preserved biomass might reflect an optimized balance between vegetative and reproductive growth under the space-limited vertical farming condition, thereby contributing to the stable yield performance of the *slga3ox2* genotype. Although *slga3ox2* plants exhibited slightly delayed fruit ripening, they produced more red fruits at harvest than the triple-determinate plants, reflecting higher fruit yield and fruit setting rate (Fig. 1L, N, Q-T). Individual fruit weight and size were similar between two genotypes grown in the vertical farm (Fig. 1U-W; Fig. S3L-M). Together, these results indicate that *slga3ox2* plants are better suited for vertical farming compared to the triple-determinate plants.

We next asked whether the maintained yield of *slga3ox2* and the reduced yield of triple-determinate in the vertical farm were associated with shifts in physiological status. We therefore applied non-destructive phenotyping for chlorophyll content (SPAD), photosynthetic efficiency (*F*_*v*_/*F*_*m*_), stomatal conductance (g_sw_), and non-photochemical quenching (*NPQ*) (Fig. S4). *slga3ox2* plants showed slightly higher SPAD values than triple-determinate plants, whereas *F*_*v*_/*F*_*m*_, g_sw_, and *NPQ* did not differ between genotypes (Fig. S4A-G). Canopy analysis further indicated a lower leaf area in *slga3ox2* plants (Fig. S4H-J). Taken together, the *slga3ox2* mutation reduces canopy size while increasing chlorophyll content per unit leaf area, with little detectable impact on core photosynthetic efficiency metrics. Under the strong spatial constraints of vertical farms (Fig. 1C), this architectural-physiological combination is consistent with improved suitability of *slga3ox2* relative to triple-determinate for sustaining fruit yield.

We demonstrate that editing *SlGA3ox2* in the triple-determinate plants produces compact, high-yielding quadruple mutants optimized for vertical farming. This phenotype contributes to increased yield per unit area (Fig. 1O), reinforcing the economic viability of vertical cultivation systems. Although yield per plant was reduced under greenhouse conditions (Fig. S3), the compact architecture of *slga3ox2* plants remains advantageous in vertical setups, where increased planting density can compensate for individual yield losses and sustain overall productivity. Finally, further trait refinement will require the implementation of advanced genome editing strategies (Rodríguez-Leal et al. 2017; Li et al. 2023, 2025).

Notably, triple-determinate plants showed a marked yield reduction under vertical farming conditions, highlighting a strong genotype-environment interaction. Microclimatic stress resulting from limited light, restricted airflow, and NFT-based hydroponic systems likely constrained performance. In contrast, *slga3ox2* mutants with compact architecture and reduced GA activity maintained stable yield, suggesting that modification of the GA pathway enhances crop resilience and productivity in vertical farming systems. Our strategies offer new opportunities to boost productivity and sustainability in controlled-environment agriculture.

## Supplementary Information

Supplementary Material 1.

Supplementary Material 2.

Supplementary Material 3.

## Data Availability

All datasets generated in this study are included in the article and supplementary information. The Sanger sequencing data for CRISPR-generated sequences are in Supplementary Material 3.
