# Supplementary material for: Designing rapid-cycling, compact architecture in tomato for vertical farming
Source: Mol Hortic. 2026 Aug 7;6:54. doi: 10.1186/s43897-026-00248-5 (PMC13450506; doi:10.1186/s43897-026-00248-5)
Supplement: Supplementary file 1 [file 43897_2026_248_MOESM1_ESM.pdf]

Supplementary information

Designing a compact, rapid-cycling tomato for vertical farming

Yoonseo Lim[1,†], Jeong-Tak An[2,†], So Jin Park[2,3], Yeeun Han[1], Ho-Young Jeong[4,5], Chanhui Lee[1,4], Soon Ju Park[6], Dae-Hyun Jung[2,3], and Choon-Tak Kwon[1,2,*]

[1] Graduate School of Green-Bio Science, Kyung Hee University, Yongin 17104, Republic of Korea.
[2] Department of Smart Farm Science, Kyung Hee University, Yongin 17104, Republic of Korea.
[3] Interdisciplinary Program in IT-Bio Convergence System, Kyung Hee University, Yongin, 17104, Republic of Korea.
[4] Department of Plant & Environmental New Resources, Kyung Hee University, Yongin 17104, Republic of Korea.
[5] Department of Seed R&D, ToolGen Inc., Cheongju 28160, Republic of Korea.
[6] Division of Applied Life Science, Plant Molecular Biology and Biotechnology Research Center, Gyeongsang National University, Jinju 52828, Republic of Korea.

*Correspondence: Choon-Tak Kwon (ctkwon@khu.ac.kr)

This PDF file includes:

Materials and methods
Supplementary references
Figures S1-S4

Materials and methods

Plant materials and growth conditions

Seeds of *Solanum lycopersicum* cv. Sweet100 were used as the initial material. Triple-determinate (*sp sp5g sler*) and *sp sp5g sler slga3ox2* gene-edited lines were generated using CRISPR-Cas9-mediated genome editing, and homozygous alleles were selected for experiments. Seeds were germinated in rockwool trays and subsequently transplanted either to greenhouse or vertical farm systems at Kyung Hee University (Yongin, Republic of Korea). In the greenhouse, seedlings were transferred 28–35 days after sowing (DAS) into ebb-and-flow hydroponic systems, following the method described in a previous study (Lim et al. 2024). The nutrient solution, renewed every three days, was maintained at pH 5.8-6.0 with electrical conductivity (EC) gradually increased from 1.0 to 1.8 $dS{\cdot}m^{-1}$. Growth was maintained under extended-day greenhouse conditions with supplemental high-pressure sodium lighting. Detailed environmental parameters of the greenhouse are provided in **Table S1**. For vertical farming experiments, seedlings were transplanted into a multi-layer nutrient film technique (NFT) system. Each cultivation tier was 26 cm in height and accommodated 16 plants, corresponding to one plant per 6,685 cm^3. Nutrient supply was delivered via a 10-min irrigation/80-min rest cycle, with EC gradually adjusted from 0.7 to 1.2 $dS{\cdot}m^{-1}$. Plants were cultivated under a long-day regime with LED lighting at 450 $\mu mol{\cdot}m^{-2}{\cdot}s^{-1}$ (16 h light/8 h dark), and environmental conditions were controlled at 24–26 °C (day), 20–22 °C (night), and 40–60% relative humidity. Plants showing symptoms of disease, nutrient deficiency, or mechanical injury were excluded from analysis.

Gene editing and plant transformation

Genome editing with the CRISPR-Cas9 system, followed by plant transformation, was carried out according to previously established protocols (Brooks et al. 2014; Kwon et al. 2020). Using the CRISPRdirect tool (https://crispr.dbcls.jp/), guide RNAs (gRNAs) were designed, and binary vectors were assembled by Golden Gate cloning according to standard protocols (Werner et al. 2012; Naito et al. 2015). For each T_0 line, genomic DNA was extracted from no fewer than three independent leaf samples to validate both the presence of the transgene and CRISPR-mediated alterations, as earlier reported (Kwon et al. 2022). Transgenic T_0 plants were transferred to soil for acclimatization, and editing results were confirmed through PCR and Sanger sequencing using conventional procedures. Details of primer sets and gRNA sequences are provided in **Table S2**.

Plant phenotyping

Phenotypic characterization was carried out on progeny lines derived from T_0 plants in which the introduced vector had segregated out over successive generations. To eliminate the possibility of residual transgenes or unintended CRISPR-induced changes, mutant tomato plants were exposed to kanamycin (300 mg/L) and tested with gene-specific primers (**Table S2**). Phenotypic evaluations were performed on non-transgenic homozygous plants obtained by self-pollination. Plant morphology, such as shoot length and total plant height, was recorded using 50-cm and 3-m rulers, respectively. Developmental characteristics, including leaf number per sympodial unit, flower number, and inflorescence count, were recorded at the appropriate growth stages. Leaf counts were taken on the main axis before the initiation of the subsequent inflorescence, consistent with standard practice. The number of biological replicates for each measurement is indicated in the figures, and detailed data are summarized in **Tables S3-S6**.

Yield trials

Yield performance of tomato plants was assessed according to established protocols with minor modifications (Lim et al. 2025). Two independent yield trials were performed using *sp sp5g sler* and *sp sp5g sler slga3ox2*$^{CR-1}$ genotypes. For uniform growth, all axillary branches were removed except for the basal axillary shoot. Yield measurements included fruits from the main stem and the retained basal axillary shoot. Fruits reaching the breaker stage were considered red. Total yield per plant was calculated as the combined weight of green and red fruits, and the harvest index was expressed as the ratio of fruit biomass to shoot biomass. The weight of individual fruits was determined with a digital balance (HANSUNG), and mature red fruit dimensions were measured with a digital caliper (Mitutoyo) and the same scale. Soluble sugar content in fruit juice was quantified as Brix (%) using a digital refractometer (ATAGO). The number of biological replicates for each analysis is presented in the figures, with numerical values compiled in **Tables S3-S6**.

Measurement of physiological parameters

Stomatal conductance to water vapor (g_{sw}) and the maximum quantum efficiency of photosystem II (F_v/F_m), obtained from chlorophyll *a* fluorescence, were determined using a portable fluorometer/porometer (LI-600, LI-COR). Leaf chlorophyll concentration was

estimated in a non-destructive manner with a SPAD meter (Konica Minolta). For each leaf, five replicate SPAD measurements were recorded, excluding major veins. To quantify photosynthetic pigments, terminal leaflets were randomly collected from fully expanded leaves. For each parameter, the number of replicates is indicated in the figures, with the associated numerical data provided both in the figures and in **Table S7**.

Chlorophyll fluorescence data acquisition

To evaluate the photosynthetic performance and stress responses of tomato plants, chlorophyll fluorescence was measured using a plant phenotyping imaging system (NITRO, PhytoWorks Inc., South Korea). The imaging resolution of the system was 1,280 × 1,024 pixels, with a field of view of 62.1° (H) × 47.7° (V). Red light (625 nm) was used as the measuring light, and blue light (450 nm) served as the saturating pulse.

Measurements were conducted according to a standardized protocol. For dark adaptation, plants were placed in the chamber for 20 min, with all internal and external LED illumination switched off to permit complete relaxation of photosystem II (PSII) reaction centers. After dark adaptation, minimal fluorescence (F_o) in the dark and maximum fluorescence (F_m) after a saturating pulse were recorded. Subsequently, during the light-adapted phase, a saturating pulse was applied to determine maximum fluorescence under steady-state conditions (F_{m_Lss}).

NPQ data calculation

Non-photochemical quenching (*NPQ*) represents the dissipation of excess excitation energy as heat and is an important indicator of photoprotective mechanisms in plants (Ruban et al. 2016). In this study, steady-state *NPQ* (NPQ_{Lss}) was calculated following the method of Cen et al. (2017), using the maximum fluorescence measured under light-adapted conditions (F_{m_Lss}), as shown in Equation (1):

$$NPQ_{Lss} = \frac{F_m - F_{m\_Lss}}{F_{m\_Lss}} \quad (1)$$

F_m denotes the maximum fluorescence measured after a saturating pulse following dark adaptation, whereas F_{m_Lss} refers to the maximum fluorescence recorded under light-adapted conditions after the application of a saturating pulse.

RGB-Depth data acquisition

Top-view RGB–Depth (RGB-D) data of training samples for constructing the depth–area regression model and tomato plant specimens were collected. Data were acquired using an Intel RealSense L515 camera (Intel Corporation, Santa Clara, CA, USA) installed in the same chamber as that used for chlorophyll fluorescence measurements. The L515 is a LiDAR-based depth sensor capable of measuring distances up to 9 m in indoor environments with a depth accuracy of ±2 mm. RGB images (1,920 × 1,080 resolution) and depth data were simultaneously recorded from the same viewpoint.

Depth-area regression model construction

A regression model was developed to convert depth information obtained from the RGB–D camera into actual area values. For calibration, nine rectangular standard objects of different sizes were prepared to cover a diverse area range. Each object was imaged from multiple heights in the top-view direction using the same RGB–D camera, and the correlation between actual area and measured depth was analyzed as follows. Background regions were first removed from RGB images using the Rembg library (v2.31.0) based on U-Net segmentation (Ronneberger et al. 2015), and masking was applied to isolate standard object regions. Because leaves exhibit irregular morphology, a pixel-based area estimation approach was adopted. Depth data corresponding to the masked RGB regions were extracted, and pixel-wise area was calculated by dividing the actual area of the rectangles by the total number of masked pixels. The average depth values for all pixels within each masked region were then computed. Linear regression analysis was performed using the actual area per pixel and the corresponding average depth values, resulting in the regression equation (2):

$$A_P = 1.1 \times 10^{-6} \times D - 3 \times 10^{-7} \tag{2}$$

A_P is the area per pixel (m^2), and D is the depth value of the corresponding pixel (m). Performance evaluation of the constructed model achieved a coefficient of determination (R^2) of 0.90 on the test dataset, demonstrating high prediction accuracy.

RGB-D-based leaf area estimation program development

A user interface for automatic leaf area estimation was developed using the PyQt5 library in a Python 3.10 environment. The system estimates leaf area through the following sequential processing steps. Users upload a folder containing RGB images and depth data acquired from

the same viewpoint. The system automatically loads both files, after which users define regions of interest (ROI) on the RGB images by rectangular mouse selection. The same ROI coordinates are then applied to the corresponding depth data. Plant regions within the ROI are segmented using an HSV (hue, saturation, value) color space–based algorithm. RGB images were first converted to HSV, and color ranges corresponding to green leaf tissues were defined. Threshold values were determined through visual inspection of tomato leaf samples as follows: hue = 27–70, saturation = 56–255, and value = 48–216. Binary masks generated from these thresholds were used to extract plant regions, which were then applied to the depth data to isolate corresponding pixel values. The regression equation (2) was applied to each pixel depth value to calculate area per pixel, and total leaf area was obtained by summing the values across all classified pixels, as shown in Equation (3):

$$A_T = \sum_{i=1}^{n} (1.1 \times 10^{-6} \times D_i - 3 \times 10^{-7}) \quad (3)$$

A_T represents the total leaf area of plant regions (m^2), n is the total number of pixels classified as plant region, and D_i represents the depth value of each pixel classified as plant region. Final results were displayed simultaneously in both m^2 and cm^2 units, and processed images from each step were visualized in real time within the user interface.

Statistical analysis

All statistical evaluations were conducted using R software (RStudio v.2022.12.0+353) and Microsoft Excel. For pairwise comparisons, significance was tested with a two-sample, two-tailed *t*-test and one-way ANOVA with Tukey test (**Table S8**).

Supplementary references

Brooks C, Nekrasov V, Lippman ZB, Van Eck J. Efficient Gene Editing in Tomato in the First Generation Using the Clustered Regularly Interspaced Short Palindromic Repeats/CRISPR-Associated9 System. PLANT Physiol. 2014;166(3):1292–7.

Cen H, Weng H, Yao J, He M, Lv J, Hua S, et al. Chlorophyll Fluorescence Imaging Uncovers Photosynthetic Fingerprint of Citrus Huanglongbing. Front Plant Sci. 2017;8:1509.

Kwon CT, Heo J, Lemmon ZH, Capua Y, Hutton SF, Van Eck J, et al. Rapid customization of Solanaceae fruit crops for urban agriculture. Nat Biotechnol. 2020;38(2):182–8.

Kwon CT, Tang L, Wang X, Gentile I, Hendelman A, Robitaille G, et al. Dynamic evolution of small signalling peptide compensation in plant stem cell control. Nat Plants. 2022;8(4):346–55.

Lim Y, Seo MG, Lee S, An JT, Jeong HY, Park Y, et al. Comparative yield evaluation of mini-tomato cultivar in two hydroponic systems. Hortic Environ Biotechnol. 2024;65(2):239–50.

Lim Y, Seo MG, Lee J, Hong S, An J, Jeong H, et al. Optimizing plant size for vertical farming by editing stem length regulators. Plant Biotechnol J. 2025;23(8):3041–53.

Naito Y, Hino K, Bono H, Ui-Tei K. CRISPRdirect: software for designing CRISPR/Cas guide RNA with reduced off-target sites. Bioinformatics. 2015;31(7):1120–3.

Ronneberger O, Fischer P, Brox T. U-net: Convolutional networks for biomedical image segmentation. Med Image Comput Comput Assist Interv. 2015;9351:234–41.

Ruban AV. Nonphotochemical Chlorophyll Fluorescence Quenching: Mechanism and Effectiveness in Protecting Plants from Photodamage. Plant Physiol. 2016;170(4):1903–16.

Werner S, Engler C, Weber E, Gruetzner R, Marillonnet S. Fast track assembly of multigene constructs using Golden Gate cloning and the MoClo system. Bioengineered. 2012;3(1):38–43.

Supplementary figure legends

Fig. S1 Predicted protein sequence and CRISPR-Cas9-induced mutations of *SlGA3ox2*. **A** Schematic representation of SlGA3ox2 protein (355 amino acids), which contains a non-haem dioxygenase in morphine synthesis N-terminal (DIOX) domain and a 2-oxoglutarate (2OG) oxygenase domain. Two CRISPR-Cas9-generated alleles harbor 1-bp insertions that introduce premature stop codons, resulting in truncated proteins lacking the C-terminal 2OG oxygenase domain. Predicted amino acid sequences of the mutant alleles are shown, with premature stop codons indicated by asterisks. **B** Shoots of triple-determinate, *slga3ox2* $^{CR-1}$, and *slga3ox2*$^{CR-2}$ plants. **C-E** Quantification of shoot length, stem internode length, number of leaves to the first inflorescence of triple-determinate, *slga3ox2* $^{CR-1}$, and *slga3ox2*$^{CR-2}$ plants. **C-E** Box plots show median, interquartile range, and whiskers; individual dots represent biological replicates. Numbers below each panel indicate sample size. Different letters between genotypes represent significant differences (P < 0.05, one-way analysis of variance and Tukey test). **B-E** Plants grown from November 2025 to February 2026.

Fig. S2 Developmental traits of triple-determinate and *slga3ox2* plants cultivated in the single-layer ebb-and-flow hydroponic system in the greenhouse. **A** Representative growth of triple-determinate and *slga3ox2* plants on the ebb-and-flow bed at 56 days after sowing (DAS). Plants were cultivated in a randomized mixed arrangement to minimize positional and environmental variation. **B–C** Plant height. **D–E** Shoot length of primary and sympodial shoots. **F** Stem internode length measured at the 5th–7th internodes. **G** Stem internode length of primary and sympodial shoots. **H–I** Leaf number up to the first inflorescence in primary and sympodial shoots. **J–K** Inflorescence number per main shoot. **L** Total number of inflorescences per plant. **B, D, F, H, J, L** Plants grown from February to July 2024. **C, E, G, I, K** Plants grown from July to October 2024. **B-L** Box plots show median, interquartile range, and whiskers; individual dots represent biological replicates. Numbers below each panel indicate sample size. Statistical significance was determined by Student's *t*-test, with P values shown in each panel.

Fig. S3 Yield performance and fruit traits of triple-determinate and *slga3ox2* plants grown in the ebb-and-flow hydroponics and vertical farm. **A, B** Representative morphology and harvested fruits of triple-determinate and *slga3ox2* plants at 95 days after

sowing (DAS). **C** Plant weight. **D** Total fruit yield per plant. **E** Proportion of red fruits relative to total yield. **F, G** Red and green fruit weight per plant. **H** Harvest index. **I** Representative fruits of triple-determinate and *slga3ox2* plants. **J, K** Individual fruit weight and size grown in the greenhouse. **L, M** Individual fruit weight and size grown in the vertical farm. **C-H, J-M** Box plots show median, interquartile range, and whiskers; individual dots represent biological replicates. Numbers below each panel indicate sample size. Statistical significance was determined by Student's t-test, with P values shown in each panel. Plants grown from July to October 2024.

Fig. S4 Non-destructive phenotypic analyses of triple-determinate and *slga3ox2* plants cultivated in the vertical farm. **A** Representative leaves of triple-determinate and *slga3ox2* plants at 68 days after sowing (DAS). **B** SPAD values. **C** Maximum quantum efficiency of photosystem II (F_v/F_m). **D** Stomatal conductance (g_{sw}). **E** Non-photochemical quenching (NPQ). **F** The plant phenotyping imaging system (NITRO, PhytoWorks Inc., South Korea) used for top-view acquisition of chlorophyll fluorescence, RGB images, and depth data. **G** Representative chlorophyll fluorescence images of tomato plants. Minimal fluorescence under dark-adapted conditions (F_0, left) and maximum fluorescence after a saturating pulse (F_m, right). **H** Raw RGB image (left) and corresponding depth map (right) acquired from the same viewpoint. **I** Screenshot of the leaf area analysis interface showing regions of interest (ROI) selection, processed depth data, segmentation masks, and calculated leaf area values in both m^2 and cm^2. **J** Leaf area estimation of triple-determinate and *slga3ox2* plants. **B-E, J** Box plots show median, interquartile range, and whiskers; individual dots represent biological replicates. Numbers below each panel indicate sample size. Statistical significance was determined by Student's *t-test*, with p values shown in each panel. Plants grown from March to June 2025.

Fig.S1

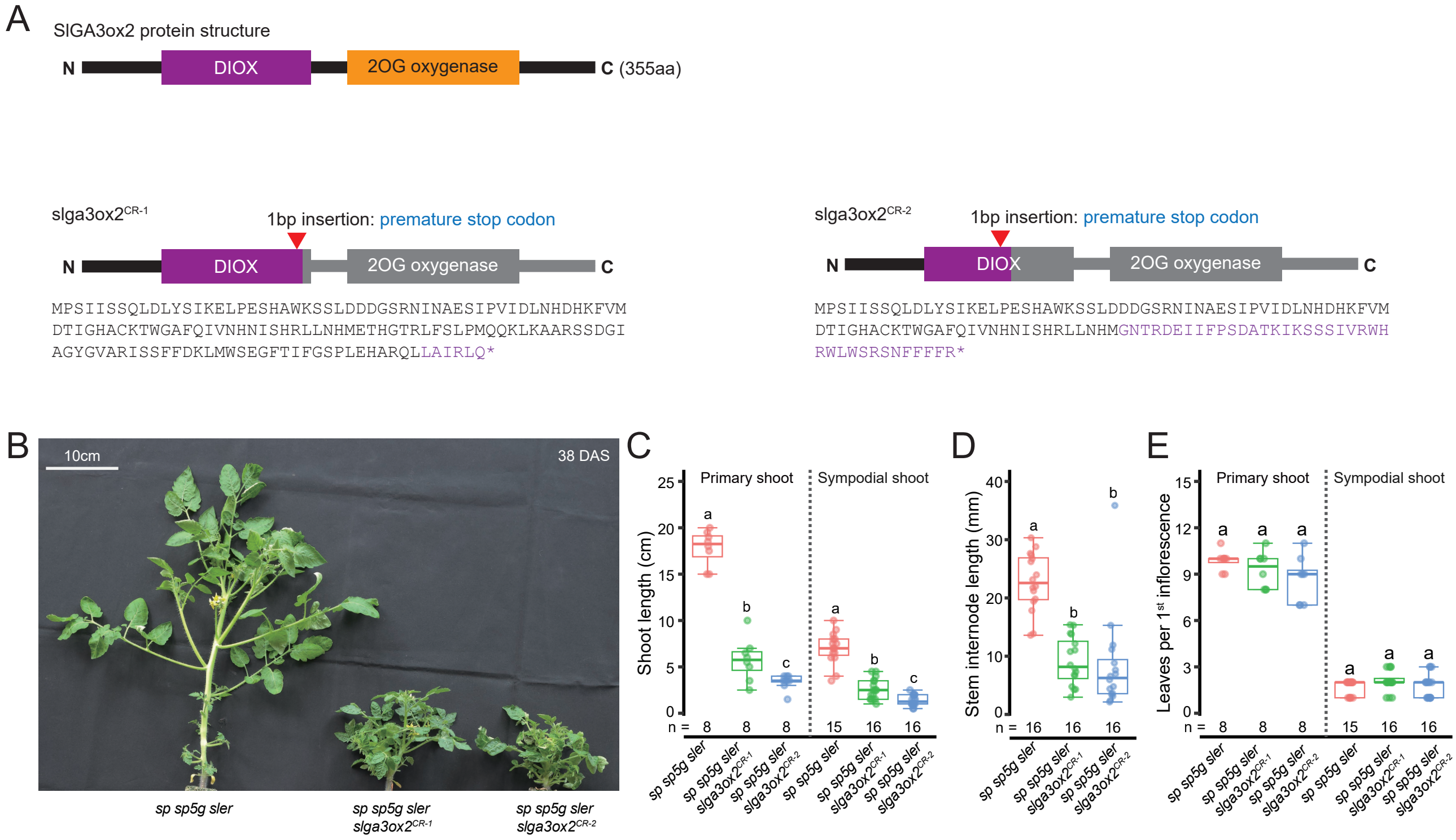

A
SlGA3ox2 protein structure
N
DIOX
2OG oxygenase
C (355aa)
slga3ox2CR-1
1bp insertion: premature stop codon
MPSIISSQLDLYSIKELPESHAWKSSLDDDGSRNINAESIPVIDLNHDHKFVM
DTIGHACKTWGAFQIVNHNISHRLLNHMETHGTRLFSLPMQQKLKAARSSDGI
AGYGVARISSFFDKLMWSEGFTIFGSPLEHARQLLAIRLQ*
slga3ox2CR-2
1bp insertion: premature stop codon
MPSIISSQLDLYSIKELPESHAWKSSLDDDGSRNINAESIPVIDLNHDHKFVM
DTIGHACKTWGAFQIVNHNISHRLLNHMGNTRDEIIFPSDATKIKSSSIVRWH
RWLWSRSNFFFFR*
B
10cm
38 DAS
sp sp5g sler
sp sp5g sler slga3ox2CR-1
sp sp5g sler slga3ox2CR-2
C
Shoot length (cm)
Primary shoot
Sympodial shoot
D
Stem internode length (mm)
E
Leaves per 1st inflorescence
n =

Fig.S2

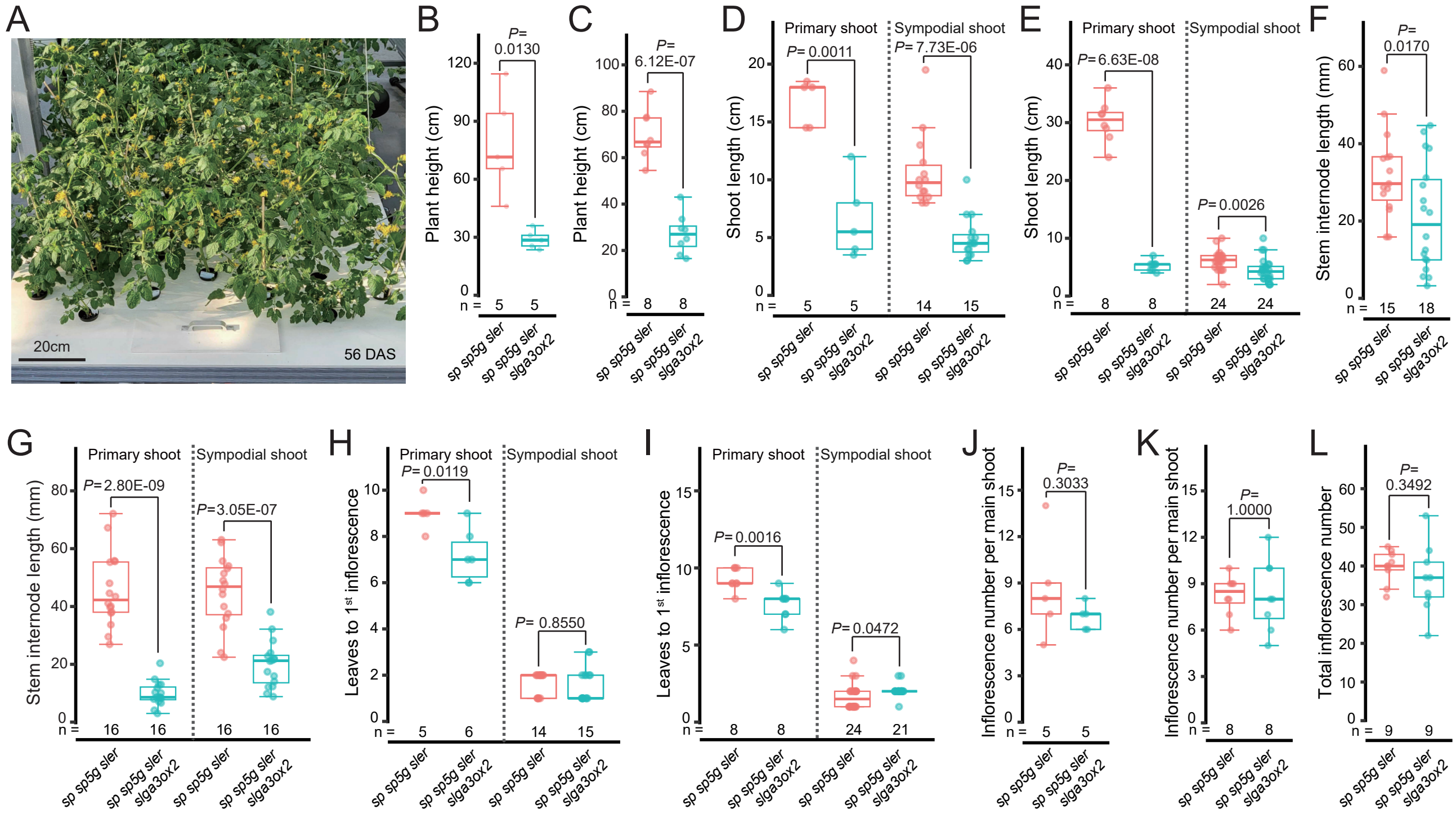

A
20cm
56 DAS
B
C
D
E
F
G
H
I
J
K
L
Plant height (cm)
Shoot length (cm)
Stem internode length (mm)
Leaves to 1st inflorescence
Inflorescence number per main shoot
Total inflorescence number
Primary shoot
Sympodial shoot
sp sp5g sler
sp sp5g sler slga3ox2

Fig.S3

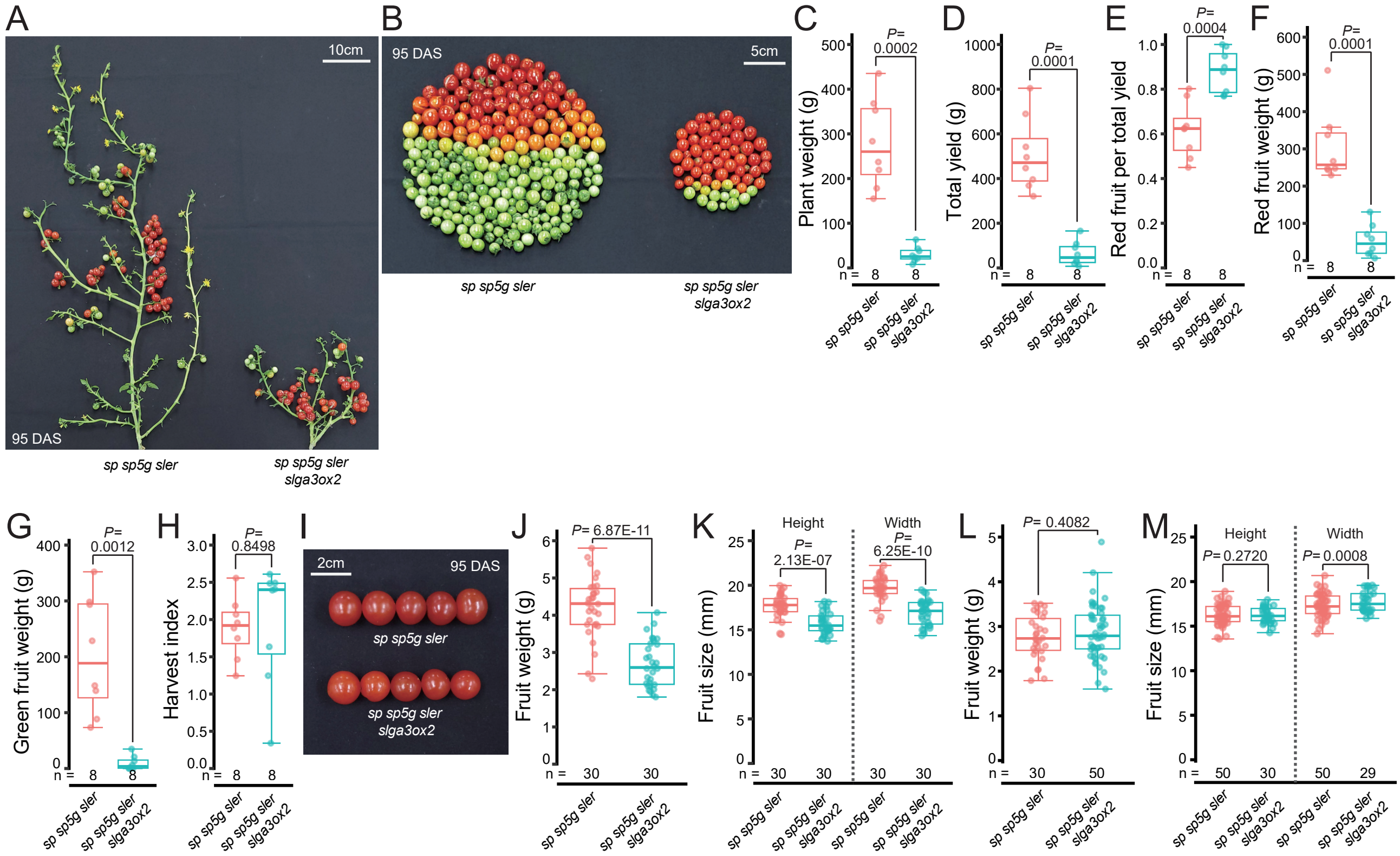
A
10cm
95 DAS
sp sp5g sler
sp sp5g sler slga3ox2
B
95 DAS
5cm
sp sp5g sler
sp sp5g sler slga3ox2
C
Plant weight (g)
P= 0.0002
D
Total yield (g)
P= 0.0001
E
Red fruit per total yield
P= 0.0004
F
Red fruit weight (g)
P= 0.0001
G
Green fruit weight (g)
P= 0.0012
H
Harvest index
P= 0.8498
I
2cm
95 DAS
sp sp5g sler
sp sp5g sler slga3ox2
J
Fruit weight (g)
P= 6.87E-11
K
Fruit size (mm)
Height
P= 2.13E-07
Width
P= 6.25E-10
L
Fruit weight (g)
P= 0.4082
M
Fruit size (mm)
Height
P= 0.2720
Width
P= 0.0008

Fig. S4

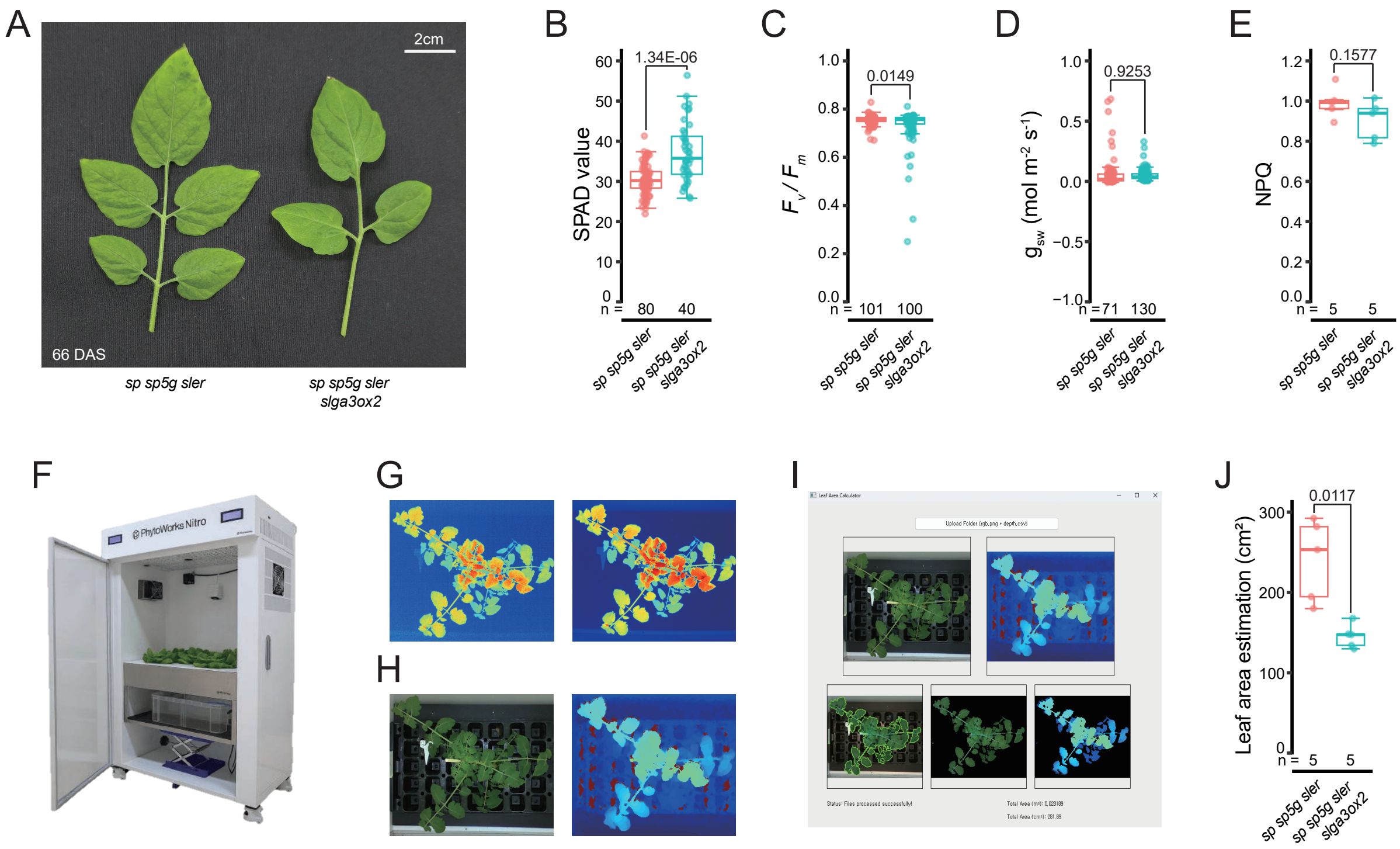

A
2cm
66 DAS
sp sp5g sler
sp sp5g sler slga3ox2
B
1.34E-06
SPAD value
n = 80 40
C
0.0149
Fv / Fm
n = 101 100
D
0.9253
gsw (mol m-2 s-1)
n = 71 130
E
0.1577
NPQ
n = 5 5
F
PhytoWorks Nitro
G
H
I
J
0.0117
Leaf area estimation (cm²)
n = 5 5
